# The Effects of Hyperbaric Oxygen Treatment on Verbal Scores in Children With Autism Spectrum Disorder: A Retrospective Trial

**DOI:** 10.7759/cureus.51654

**Published:** 2024-01-04

**Authors:** Tami Peterson, Robert Sherwin, Tiffany Hosey, Nicole Close, Frederick Strale Jr.

**Affiliations:** 1 Hyperbaric Oxygen Therapy, The Oxford Center, Brighton, USA; 2 Hyperbaric Oxygen Therapy, Wayne State University School of Medicine, Detroit, USA; 3 Biostatistics, EmpiriStat, Kitty Hawk, USA; 4 Biostatistics, The Oxford Center, Brighton, USA

**Keywords:** assessment placement program, autism spectrum disorder (asd), hyperbaric oxygen therapy (hbot), retrospective trial, verbal behavior milestones

## Abstract

Introduction

Autism spectrum disorder (ASD) is a neurodevelopmental condition that affects millions worldwide. Suggested pathophysiology includes cerebral hypoperfusion, inflammation, mitochondrial and immune dysregulation, and oxidative stress. Debate exists concerning the benefit of hyperbaric oxygen therapy (HBOT) in treating ASD and its impacts on verbal behavior. The present study directly assesses the impacts of HBOT treatments on verbal behavior using a novel and unique manner.

Materials and methods

A two-group quasi-experimental trial using a pretest and a posttest was designed to retrospectively assess (n = 65) any association between HBOT and change in verbal scores in children (n = 65) with ASD. All children completed two verbal tests six months apart, either the Verbal Behavior Milestones Assessment and Placement Program (VBMAPP) or the Assessment of Basic Language and Learning Skills (ABLLS), based on their developmental age. The control cohort received applied behavior analysis (ABA) without HBOT. The experimental cohort received ABA and a minimum of 40 HBOT treatments, breathing 100% oxygen at 2.0 atmosphere absolute (ATA) for 60 minutes.

Results

Sixty-five children were included, of which 32 received HBOT (mean (M) = 5.1, standard deviation (SD) = 2.93), with an age range of two to 17 years. More than 63% of the subjects had an autism severity level of three. The 23 children administered VBMAPP who received HBOT showed substantial mean differences with high effect sizes (ESs) (-0.743 to -1.65) and a total score (TS) ES equal to -1.23 as measured by Cohen's d. There was a statistically significant improvement (p < 0.05) in all VBMAPP milestone domains and TS. TS change from baseline versus those in the non-HBOT (Control-ABA) group (n=12) was 46.41 ± 20.14 vs 14.42 ± 6.99; p < 0.0001, ES = -1.23. The 30 children administered the ABLLS showed substantial mean difference (TS) change from baseline 268.89 ± 182.05 vs 190.81 ± 135.26 and exhibited small to medium (-.114 to -.773) ESs with a TS ES = -0.487. Due to the high within-group variability (low statistical power) within the ABLLS cohort, there was a non-significant mean difference between the control (ABA) and experimental (ABA + HBO2) groups' difference scores (p > 0.2024), despite the medium (TS) ES.

Conclusions

The child cohorts administered the VBMAPP and the ABLLS demonstrated substantial improvements between the non-HBOT (control-ABA) and HBOT (experimental-ABA + HBO2) groups as measured by the significant mean differences and small to large ESs. Simply put, the children in the experimental cohort acquired more verbal skills than their counterparts in the control group.

## Introduction

Autism spectrum disorder (ASD) is a neurodevelopment disorder that involves difficulties in verbal and social interactions, repetitive behaviors, decreased communication skills, environmental interaction, literacy impairments, and general interests. Impairments in spoken language are common. The Diagnostic and Statistical Manual of Mental Disorders (DSM-5) lists several diagnostic criteria for autism, including a lack of social and verbal communication, reduced social and verbal interaction across multiple contexts, and restrictive or repetitive patterns of behavior that are present early in development and cause significant impairment in social, occupational functioning not otherwise explained by another comorbidity [1]. ASD adheres to no socioeconomic, racial, or ethnic boundaries but has been reported to be far more prevalent in boys than girls. It impacts the life of everyone involved. In up to a third of cases, there can be a regression of developmental achievements early in life [2]. According to the Centers for Disease Control and Prevention (CDC), the current prevalence (in eight-year-old children) is one in 54, up from one in 59 in 2018, and has continued to rise since the CDC began reporting on autism in 2000 [3]. 

The contemporary hypothesis underlying ASD is that genetic predisposition and environmental exposures may lead to its development [4]. Up to 1,000 genes may be involved in developing ASD [5]. The pathophysiological basis of ASD is debated but includes oxidative stress, cerebral hypoperfusion, inflammation, mitochondrial dysfunction, and immune dysregulation [6,7,8,9]. Cerebral hypoperfusion, oxidative stress, and neuroinflammation have been described in multiple studies of ASD patients and offered as targets for the potential benefit of hyperbaric oxygen therapy (HBOT) [8,9,10,11]. 

Regarding verbal behavior, some autistic children may be unable to communicate using speech or language, and some may have minimal speaking skills, resulting in difficulty expressing themselves and understanding what others are saying. Some children with ASD may have highly developed vocabulary skills and have detailed conversations about various topics but may struggle using verbal behavior in social circumstances, such as initiating conversations, greeting people, and asking for things [12]. They may also have facial expressions, eye contact, and body language difficulties. Some children with autism may repeat other people’s words (echolalia) immediately or later. Reading, writing, spelling, and comprehension are common literacy impairments affecting academic performance, information access, and writing abilities [12]. Verbal deficits in children with ASD may also include sensory issues. They may be hypersensitive to sounds and loud noises, which may cause them to avoid speech, or they may be hyposensitive to sounds and loud noises, which may make them unaware of the volume of their voice [12]. 

Given this, there are various treatment options for ASD, including behavioral, developmental, educational, social-relational, pharmacologic, psychological, and complementary or alternative interventions. Unfortunately, despite early diagnosis and intensive therapy, patients with ASD still experience significant negative impacts on social and verbal interaction, academic, and overall life experience [10]. As a result, many parents pursue various therapeutic options, including melatonin, secretin therapy, diet modification, vitamin supplements, and HBOT [10]. HBOT involves the delivery of 100% oxygen at pressures above atmosphere. HBOT has been shown to influence many physiologic levels, including decreasing inflammation, mitochondrial function, correcting tissue hypoxia, and augmenting the body’s ability to handle oxidative stress. These benefits of HBOT overlap with several theories of the pathophysiologic basis of ASD [13]. 

The Cochrane Database and the Agency for Healthcare Research and Quality (AHRQ) reviews on this topic could not provide a definitive recommendation with regard to the clinical question as to the impacts of HBOT treatments on verbal behavior. Many publications either utilize interventions that do not meet Undersea and Hyperbaric Medical Society (UHMS) definition of hyperbaric oxygen or lack adequate control groups or appropriate methodology. 

There have been multiple literature and systematic reviews concerning the use of HBOT in treating ASD symptoms, which have reported mixed results, ranging from no benefit to some reporting promising results [11]. A barrier to a comprehensive understanding of this body of literature is that it is very heterogeneous due to the defined outcomes, differing patient populations, comparator groups (use of sham treatment versus control or none), and the selected pressure and oxygen level administered to the treatment groups. Despite the body of literature that addresses the efficacy of HBOT in ASD, research is still warranted due to the scarcity of well-performed trials [13,14,15,16,17,18]. This study is novel and unique as it directly assesses the effects of HBOT treatments on verbal behavior using unique and novel measures. There is a gap in the literature relative to this, and the present study aims to fill that gap. 

Objectives 

The primary objective and outcome of interest of this study is to evaluate any association between HBOT, and the improvement of verbal skills measured by either Verbal Behavior Milestones Assessment and Placement Program (VBMAPP) [12] or the Assessment of Basic Language and Learning Skills (ABLLS) [14] in a convenience cohort of children with ASD. The secondary objective of the trial was to assess the safety of HBOT in this patient population. The specific verbal testing administered was based on the child’s developmental age. It is hypothesized that the group receiving the HBOT treatment (the experimental group) will show increased verbal capabilities compared to the group not receiving the HBOT.

## Materials and methods

Study setting and participants

All autistic child cohorts were recruited, seen, and treated at The Oxford Centers (TOCs), in Brighton and Troy, Michigan, USA, between January 2018 and July 2021 (43 months). TOCs are outpatient facilities that provide various clinical services for several conditions, including ASD. These services include but are not limited to applied behavioral analysis (ABA) therapy, nutrition therapy, neurofeedback, musical therapy, educational support, HBOT, and physical, occupational, and speech therapy. Children treated at TOC can receive any of these therapies and may or may not receive HBOT based on parental discretion. 

Data collection

Study data were collected retroactively from electronic medical records by trained research assistants to gather data relative to ABA child cohort patients treated with either non-HBOT (control-ABA) or the HBOT (ABA + HBO2) treatment. Three different "non-author" Board-Certified Behavior Analysts (BCBAs) collected the original pretest and posttest VBMAPP and ABLLS data for both control and experimental groups. Both groups received ABA treatments. Manuscript generation and reporting adhered to the Consolidated Standards of Reporting Trials (CONSORT) and Strengthening the Reporting of Observational Studies in Epidemiology (STROBE) guidelines. 

Records of children cohorts aged two to 17 years diagnosed with ASD and treated between January 2018 and July 2021 (48 months) were screened for inclusion in this study. Each child who received HBOT had completed a minimum of 40 sessions at 2.0 atmosphere absolute (ATA) during the study period and had two or more age-appropriate verbal tests conducted by a qualified practitioner. A BCBA also administered children not receiving HBOT the VBMAPP and ABLLS verbal tests. Any child diagnosed with a seizure disorder or genetic or mitochondrial mutation was excluded from the study cohort. Those who received HBOT were included in the treatment group, and those who did not receive HBOT were included in the control group.

Matched pairs were created (pretest-posttest), so children's cohorts shared every characteristic except the HBOT intervention. A total of five males were excluded from the study in the HBOT treatment group because the initial verbal test was given after the first HB02 session (n = 3), the post-test was given before the last HB02 session (n = 1), and the verbal test was missing data (n = 1). Three males and three females were excluded from the non-HBOT control group for not meeting the inclusion criteria because of incomplete assessments (n = 2), duplicate entry (n = 1), and missing data (n = 3). The children cohorts served as their own controls in the pretest-posttest, thus reducing potential bias.

Interventions

HBOT Sessions

HBOT sessions were conducted in the Class B chamber approved by the Food and Drug Administration (FDA). They can treat patients between 1 and 3 ATA with an average oxygen percentage of 99.803%, confirmed and verified through a gas analysis by a third party. The monoplace hyperbaric chamber model used in this trial was a Sechrist 3300H (Sechrist Industries, Inc., Anaheim, California). The patients in the HBOT group (n = 32) were pressurized with medical-grade oxygen to 2.0 ATA at a rate of 1-2 pounds per square inch per minute (psi/min) for five sessions per week. The subject would breathe the oxygenated environment at a treatment depth for 60 minutes without air breaks, while being monitored for adverse events (AEs) by a trained hyperbaric technician. The hyperbaric chamber was depressurized at a rate of 1-2 psi/min back to 1.0 ATA. 

AEs

AEs were collected only for the treatment group subjects as related to HBOT. All verbatim AE terms were coded using the Medical Dictionary for Regulatory Activities (MedDRA) and summarized by System Organ Class (SOC) and Preferred Term (PT). AE collection began from the start of the study period through the end of the study period. AEs were summarized overall by the number and percentage of subjects who experienced at least one AE. When AEs were reported, the hyperbaric technician would report the AE to an attending nurse and supervising physician.

ABA Procedure

All patients received ABA treatment, which is a control variable and is held constant. Patients in both the control and experimental groups received the same number of treatments. All patients received a minimum of 25 ABA treatment hours per week. ABA is a one-on-one therapy technique that develops the skills that make clients more successful in their home, school, and communities through natural environment teaching and discreet trial teaching. 

Outcome measures

A BCBA administered the initial assessment based on their developmental age, either with VBMAPP or ABLLS. Preliminary goals were set and then re-evaluated after an observation period. The VBMAPP and ABLLS were administered to both the control non-HBOT and experimental HBOT groups at baseline (pretest) and then again after the 40th HBOT session, at least 40 sessions for 60 minutes at 2.0 ATA duration, if applicable. Control for potential confounding variables was accounted for by matched pairs (pretest-posttest) whereby each child cohort served as their own control. Changes in medications and started/stopped other therapies, such as speech therapy, physical therapy, and occupational therapy, were recorded.

VBMAPP

Child cohorts (n = 23) were assessed by the BCBA at pretest and posttest in the HBOT treatment group and non-HBOT + ABA control group (n = 12). Each child cohort was tested on behavioral milestone domains: mand, tact, listener, visual/perceptual skills, independent play, social, motor Imitation, echoic, listener responding, intraverbal, group behavior, and linguistic structure. These skills are necessary for the acquisition of language and social skills. Each child cohort is observed, prompted, and assigned a rating on a five-point Likert scale grid based on response behaviors recorded by the BCBA. The higher the score on the milestone sub-scale, the better the child cohort’s progress [15]. Cronbach's alpha on VBMAPP for this sample was r = 0.936 [18].

ABLLS

Child cohorts (n = 30) were assessed on a grid by a BCBA at pretest and posttest in the HBOT treatment group (n = 9) and non-HBOT control group (n = 21). Each child cohort was tested on behavioral milestone subscales: receptive, requests, labeling, intraverbals, spontaneous vocalizations, syntax grammar, social interaction, and generalized responding [16]. Cronbach's alpha on ABLLS for this sample was r = 0.869 [18].

Sample size determination

A retrospective power analysis was conducted using G*Power 3.1 [17] and indicated that a total sample size of n = 64 participants would be required to demonstrate a high group effect size (ES) (0.80) for the two groups' mean comparisons (HBOT vs. non-HBOT) with nominal alpha (α) = 0.05 using a two-tailed two independent sample t-test, with a power equal to 0.882. Given these power analysis parameters, there is a high likelihood that this current retrospective trial with n = 65 participants satisfies an acceptable sample size criterion. No other studies were used to estimate the sample size.

Statistical methods 

IBM SPSS Statistics for Windows, version 29 (released 2022; IBM Corp., Armonk, New York, United States) [18] was used for all descriptive and inferential data analyses. Demographics and baseline characteristics were summarized for all subjects overall and by treatment.

Summary statistics (e.g., number of subjects, mean and standard deviation, and median and range) were generated for all continuous variables, i.e., age, HBOT treatment months, months from the baseline verbal test to the post baseline verbal test, months from the baseline verbal test to the first HBOT treatment, months from the last HBOT treatment to the post-baseline verbal test, age VBMAPP, age ABLLS, and the number and percentage of subjects within each category, were presented for all categorical variables (i.e., race/ethnicity and autism severity level).

The change from baseline in the VBMAPP and ABLLS scores were summarized along with the incidence of adverse and serious events by treatment group. All components of the oral tests were summarized and presented with descriptive statistics by subject and study treatment. The mean change from baseline in the overall verbal test score was compared between those who received hyperbaric treatment and those who did not using a two independent sample t-test with a two-tailed scenario using an alpha (α) of 0.05. Statistical significance was declared for p < 0.05. All statistical results were reported via text and table presentation.

Institutional Review Board (IRB)

This research study was conducted retrospectively from data obtained via chart review for clinical purposes. The study was submitted to the WIRB-Copernicus Group (WCG® IRB) for review and received an exemption (#1-1435713). The authors hereby certify that the analysis was performed in accordance with the ethical standards as put forth in the 1964 Declaration of Helsinki [19] and its later amendments or comparable ethical standards. Please note that since obtaining the ClinicalTrials.gov Identifier: NCT06043284, Oxford Recovery Center (ORC) has changed its name to The Oxford Center (TOC) (other study ID numbers: OxRS-01-2021).

## Results

Table 1 indicates that there were 65 children cohorts included in the trial, of which 32 children cohorts received hyperbaric treatment (31 males and one female) and 33 children cohorts did not (27 males and six females). Baseline characteristics were comparable as all p-values were non-significant (p > 0.05). The statistical equality between the HBOT (experimental) vs. non-HBOT (control) groups is presented in Table 1 using p-values, as all analyses were non-significant (p > 0.05). On average, the age of children cohorts was (M = 5.7, SD = 3.08) with a range of two to 17 years. 

**Table 1:** Baseline characteristics of the HBOT (ABA + HBO2) and non-HBOT (ABA only) groups Two independent-sample t-tests, two-way chi-squares, and Fisher’s exact tests were used at α = 0.05. Statistical significance was achieved at p < 0.05. ABA: applied behavior analysis, HBOT: hyperbaric oxygen therapy

Characteristics	Statistical procedure	HBOT group (ABA + HBO2) (n = 32)	Non-HBOT group (ABA only) (n = 33)	Total (n = 65)	p-value
Treatment months	Mean	5.53		5.53	N/A
	SD	1.08		1.08	
	Median	6		6	
	Min	2		2	
	Max	6		6	
Months from the baseline verbal test to post-baseline verbal test	n	32	33	65	
	Mean	5.53	5.8	5.7	0.215
	SD	1.08	0.73	0.80	
	Median	6	6	6	
	Min	2	4	2	
	Max	6	8	8	
Months from the baseline verbal test to the first HBOT treatment	n	32	0	32	
	Mean	1.0		1.0	N/A
	SD	1.22		1.22	
	Median	0		0	
	Min	0		0	
	Max	4		4	
Months from the last HBOT treatment at post-baseline verbal test	n	32	0	32	
	Mean	2.81		2.81	N/A
	SD	1.40		1.40	
	Median	3		3	
	Min	0		0	
	Max	5		5	
Age (years)	n	32	33	65	
	Mean	5.1	6.55	5.7	0.084
	SD	2.93	3.58	3.08	
	Median	4	5	5	
	Min	2	2	2	
	Max	17	16	17	
Age (VBMAPP) years	n	23	12	35	
	Mean	3.96	4.08	5.7	0.744
	SD	1.07	1.08	3.08	
	Median	4	5	5	
	Min	2	2	2	
	Max	17	16	17	
Age (ABLLS) years	n	9	21	30	
	Mean	8.1	7.96	7.7	0.921
	SD	4.01	3.76	3.46	
	Median	8	7	7	
	Min	3	4	3	
	Max	17	16	17	
Race/ethnicity, n (%)	African	1 (3.1)	0 (0.0)	1 (1.5)	0.670
	American Indian	0 (0.0)	1 (3.0)	1 (1.5)	
	Asian	1 (3.1)	0 (0.0)	1 (1.5)	
	Hispanic	4 (12.5)	3 (9.1)	7 (10.8)	
	Middle Eastern	3 (9.4)	2 (6.1)	5 (7.7)	
	White	18 (56.3)	23 (69.7)	41 (63.1)	
	American Indian	0 (0.0)	1 (3.0)	1 (1.5)	
	Unspecified	5 (15.6)	4 (12.1)	9 (13.8)	
Autism severity level n (%)	1	1 (3.1)	3 (9.1)	4 (6.2)	0.495
	2	9 (28.1)	11 (33.3)	20 (30.8)	
	3	22 (68.8)	19 (57.6)	41 (63.1)	
Gender (%)	Male	31 (96.9)	27 (81.8)	58 (89.2)	0.103
	Female	1 (3.1)	6 (8.2)	7 (10.8)	

Over 63% of child cohorts had an autism severity level of 3. In comparison, almost 31% had a severity level of 2, and over 6% were level 1. A considerable percentage (63.1%) were classified as Middle Eastern, and 30% were classified as Caucasian. In the control group (non-HBOT), 12 had been administered VBMAPP, and 21 were administered ABLLS. For those receiving HBOT, the intervention was delivered over an average of 5.53 months (SD = 1.08), ranging from two to six months. Age-appropriate verbal testing was conducted, where 35 children (M = 5.7 years old, SD = 3.08) were administered the VBMAPP (ages two to 17 years) and 30 children (M = 7.7 years old, SD = 3.46) were administered the ABLLS (ages three to 17 years). In the HBOT treatment group, 23 children (M = 3.96 years old, SD = 1.07) were administered VBMAPP while n = 9 (M = 8.1 years old, SD = 4.01) were administered ABLLS. 

For the patients evaluated by VBMAPP (see Table 2), there were substantial mean differences observed, with large (-0.743 to -1.650) ESs and a total score ES = -1.23. There were statistically significant differences (p < 0.05) between all baseline and post-baseline difference scores between the control and treatment groups. 

**Table 2:** VBMAPP change in difference scores between the control (ABA) and experimental (ABA + HBO2) groups Two-independent sample t-tests and Cohen's d were used at α = 0.05. Statistical significance was achieved at p < 0.05. ABA: applied behavior analysis, HBO2: hyperbaric oxygen, VBMAPP: Verbal Behavior Milestones Assessment and Placement Program, SD: standard deviation, CI: confidence interval

Scale	Time	HBOT (ABA + HBOT) Mean (SD) n = 24	Non-HBOT (ABA only) Mean (SD) n = 13	Mean difference	95% (CI)	SE	Effect size (d)	95%(CI)	p-value
Mand	Pretest	2.41 (3.34)	2.96 (2.85)						
	Posttest	6.43 (4.00)	4.33 (2.82)						
	Difference	4.02 (2.37)	1.38 (0.83)	2.65	1.53, 3.77	0.55	-1.33	-2.09, -0.553	<0.0001
Tact	Pretest	2.24 (3.02)	3.00 (2.88)						
	Posttest	6.50 (4.11)	4.17 (3.03)						
	Difference	4.26 (2.96)	1.17 (1.05)	3.09	1.69, 4.50	0.69	-1.24	-1.99, -0.474	<0.0001
Listener responding (LR)	Pretest	3.20 (3.10)	3.92 (3.64)						
	Posttest	7.50 (4.03)	5.71 (3.31)						
	Difference	4.30 (2.42)	1.79 (1.41)	2.51	0.96, 4.06	0.76	-1.18	-1.92, -0.416	0.0023
Visual perceptual skills and matching-to sample (VP-MTS)	Pretest	4.57 (2.77)	5.38 (3.49)						
	Posttest	7.98 (3.65)	6.79 (3.12)						
	Difference	3.41 (1.86)	1.42 (1.13)	2.00	0.80, 3.19	0.59	-1.21	-1.96, -0.444	0.0018
Independent play	Pretest	4.22 (3.92)	5.63 (2.82)						
	Posttest	8.26 (4.10)	6.96 (2.56)						
	Difference	4.04 (2.75)	1.33 (1.37)	2.71	1.29, 4.13	0.70	-1.14	-1.88, -0.382	0.0005
Social play	Pretest	2.22 (2.27)	2.88 (2.14)						
	Posttest	6.22 (2.68)	4.50 (2.29)						
	Difference	4.00 (1.97)	1.63 (1.19)	2.38	1.11, 3.64	0.62	-1.36	-2.12, -0.581	0.0006
Motor imitation	Pretest	1.93 (2.44)	2.29 (2.21)						
	Posttest	6.24 (2.93)	4.00 (2.44)						
	Difference	4.30 (2.44)	1.71 (1.27)	2.60	1.32, 3.87	0.63	-1.22	-1.97, -0.458	0.0002
Echoic	Pretest	1.65 (2.34)	4.08 (4.25)						
	Posttest	5.98 (3.40)	4.29 (4.16)						
	Difference	4.33 (3.04)	0.21 (0.33)	4.12	2.79, 5.45	0.64	-1.65	-2.46, -0.840	<0.0001
Spontaneous vocalization	Pretest	1.93 (1.73)	2.42 (1.74)						
	Posttest	4.96 (2.50)	3.13 (1.57)						
	Difference	3.02 (2.28)	0.71 (0.78)	2.31	1.24, 3.39	0.53	-1.21	-1.96, -0.445	0.0001
Listener responding by function, feature, and class (LRFFC)	Pretest	0.57 (1.23)	0.67 (2.02)						
	Posttest	3.28 (2.71)	1.13 (2.07)						
	Difference	2.72 (2.13)	0.46 (0.94)	2.26	1.20, 3.32	0.52	-1.24	-1.99, -0.475	0.0001
Intraverbal	Pretest	0.35 (0.83)	0.58 (1.29)						
	Postest	2.72 (2.40)	1.46 (1.66)						
	Difference	2.37 (1.86)	0.88 (0.93)	1.49	0.53, 2.46	0.47	-.928	-1.63, -0.189	0.0033
Group behavior	Pretest	0.89 (1.87)	2.13 (2.25)						
	Posttest	4.67 (2.29)	3.25 (1.75)						
	Difference	3.78 (1.92)	1.13 (1.32)	2.66	1.39, 3.92	0.62	-1.52	-2.30, -0.726	0.0002
Linguistic structure	Pretest	0.74 (1.57)	1.00 (1.71)						
	Posttest	2.59 (2.64)	1.63 (2.43)						
	Difference	1.85 (1.91)	0.63 (0.91)	1.22	0.25, 2.19	0.48	-.743	-1.46, -0.018	0.0151
Total score	Pretest	26.91 (27.06)	36.92 (27.48)						
	Posttest	73.33 (35.19)	51.33 (26.04)						
	Difference	46.41 (20.14)	14.42 (6.99)	32.00	22.44, 41.51	4.66	-1.23	-1.91, -0.548	<0.0001

For patients evaluated by ABLLS (see Table 3), there were substantial mean differences observed, with small to medium (-0.114 to -0.773) ES and a total score ES = 0.487. However, there were no statistically significant differences (p = 0.2024) between the baseline and post-baseline difference scores between the treatment groups. This is attributed to a low statistical power relative to the high within-group variation between the control and experimental groups.

**Table 3:** ABLLS change in difference scores between the control (ABA) and experimental (ABA + HBO2) groups Two independent-sample t-tests and Cohen's d were used at α = 0.05. Statistical significance was achieved at p < 0.05. ABLLS: Assessment of Basic Language and Learning Skills, ABA: applied behavior analysis, HBO2: hyperbaric oxygen, SD: standard deviation, CI: confidence interval

Scale	Time	HBOT mean (SD) n = 24	Non-HBOT mean (SD) n = 13	Mean difference	95% CI	SE	Effect size (d)	95% CI	p-value
Receptive language	Pretest	76.56 (37.26)	88.71 (56.12)						
	Posttest	134.78 (47.22)	134.29 (63.15)						
	Difference	58.22 (43.18)	45.57 (33.23)	12.65	-17.00, 42.20	14.47	-0.348	-1.13, 0.441	0.3895
Requests	Pretest	52.22 (24.67)	36..00 (27.47)						
	Posttest	96.22 (36.75)	61.00 (34.39)						
	Difference	44.00 (31.13)	25.00 (21.38)	19.22	-1.05, 39.05	9.79	-0.773	-1.57, 0.039	0.0623
Labeling	Pretest	52.89 (43.49)	49.33 (48.82)						
	Posttest	99.56 (55.07)	86.48 (61.90)						
	Difference	46.67 (43.83)	37.14 (31.46)	9.52	-19.40, 38.44	14.12	-0.269	-1.05, 0.518	0.5055
Intraverbal	Pretest	42.44 (35.21)	33.48 (36.07)						
	Posttest	93.00 (55.10)	67.48 (55.04)						
	Difference	50.56 (40.47)	34.00 (36.71)	15.56	-14.31, 47.42	15.07	0.438	-1.22, 0.355	0.2813
Spontaneous vocalizations	Pretest	23.89 (12.13)	20.71 (13.07)						
	Posttest	30.11 (8.19)	28.00 (10.19)						
	Difference	6.22 (11.68)	7.29 (8.21)	-1.06	8.68, 6.55	3.72	-0.114	-0.668, 0.894	0.7769
Syntax and grammar	Pretest	18.44 (15.83)	12.90 (21.39)						
	Posttest	37.78 (28.87)	24.67 (25.22)						
	Difference	19.33 (16.39)	11.76 (17.92)	7.57	-6.71, 21.85	6.97	-0.433	-1.22, 0.360	0.2867
Social interactions	Pretest	41.22 (26.07)	28.05 (20.33)						
	Posttest	76.33 (40.86)	50.33 (27.71)						
	Difference	35.11 (25.97)	21.00 (22.29)	12.83	-4.24, 29.89	8.33	-0.613	-1.41, 0.189	0.1349
Generalized responding	Pretest	7.78 (7.10)	9.76 (8.61)						
	Posttest	16.56 (9.50)	17.52 (7.90)						
	Difference	8.78 (7.45)	7.76 (6.04)	1.02	-4.27, 6.30	2.58	-0.157	-0.937, 0.626	0.6966
Total score of the assessment of language and basic living skills	Pretest	315.44 (154.65)	278.95 (197.74)						
	Posttest	584.33 (238.25)	469.76 (256.28)						
	Difference	268.89 (182.05)	190.81 (135.26)	78.08	-44.43, 200.59	59.81	-.487	-1.14, 0.369	0.2024

There were no reported serious adverse events (SAEs) during the study period for the treatment group. There were 11 adverse events reported in six treatment group subjects during the HBOT session. AEs reported included ear discomfort (12.5%), vomiting (3.1%), dyspnea (3.1%), and sinus congestion (3.1%). All AEs were grade 1 (mild), and all but two of the events were determined to be related to HBOT. The two non-HBOT AE children may have been experiencing autistic symptoms that could not be remedied because of the nature of their autism symptoms.

## Discussion

HBO2 increases oxygen tension, which increases oxygen content within the tissues, assisting with protecting the circulatory system down to the capillary level. Since air pressure increases two to three times that of normal air pressure within the HBO2 chamber, hyperoxygenation occurs within the lungs that would not usually occur at normal air pressure. This expanded oxygen supply positively impacts widely varied medical and psychological conditions. Adding oxygen to hypoxic tissues and resulting hyperoxemia in the blood helps increase neovascularization and reduces or eliminates infections [20-25].

Statistically significant differences (p < 0.05) were found between the HBOT treatment group and the non-HBOT control group for each VBMAPP domain: mand, tact, listener, visual/perceptual skills, independent play, social, motor imitation, echoic, listener responding, intraverbal, group behavior, and linguistic structure and the total score. These skills are necessary for the acquisition of language and social skills. 

For patients evaluated by ABLLS, despite noteworthy mean differences between the non-HBOT control and HBOT treatment groups (see Table 3), there were no statistically significant differences between the baseline and post-baseline difference scores. This statistical phenomenon resulted from the low statistical power due to a sizable within-group variability. However, there were small to medium ESs, as reported by Cohens' d.

ASD affects millions of children worldwide with rising incidence. Despite many therapies, affected individuals remain at significant risk for poor social, academic, and developmental outcomes. The pathophysiologic basis of ASD is not entirely understood, but evidence supports the contribution of oxidative stress [26,27], mitochondrial dysfunction [27], neuroinflammation [4], immune dysregulation [4,26], and cerebral hypoperfusion [4,11]. The current literature is inconclusive, primarily due to the lack of well-controlled trials. 

In this study of 65 children with ASD, patients who were evaluated with VBMAPP had significantly improved scores in the HBOT vs. non-HBOT groups. Patients assessed by ABLLS showed noteworthy mean differences between the non-HBOT control and HBOT treatment groups and a medium ES but did not show significance. The intervention in this study satisfies the current definition of HBOT per the UHMS. Both VBMAPP and ABLLS are widely used and accepted assessments for ASD patients and are valid outcome measures.

A Cochrane Review in 2016 included only one trial. It concluded that no current evidence exists that HBOT improved social interaction, behavioral problems, speech or language communication, or mental function in children with ASD [28,29]. The included trial [28] reported a randomized controlled trial on ages three to nine years who were randomized to 20x one-hour HBOT sessions at 1.5 ATA or sham air at 1.15 ATA. Although overall behavior improved in both arms, there was no significant benefit of HBOT over sham.

Granpeesheh et al.'s study [29] was excluded because the intervention, given at 1.3 ATA and 24% oxygen, did not meet the definition of HBOT, which mandated an ATA greater than 1.4 and a 100% oxygen level. Rossignol et al.'s study [28] was notable as it was the first controlled trial evaluating HBOT use in ASD. However, it did not meet the definition of HBOT, which mandated an ATA greater than 1.4 and a 100% oxygen level. Nineteen studies were excluded from the literature review for this study as they did not meet the definition of HBOT, which mandated an ATA greater than 1.4 and a 100% oxygen level.

An AHRQ review of medical therapies for ASD included four studies [26,27,28,29]. This review included studies that did not necessarily meet the UHMS definition of HBOT administration criterion, 1.5 ATA and 100% oxygen, and were excluded from the 2016 Cochrane paper. This review concluded that the current data were inadequate to determine the efficacy of HBOT in ASD, but also noted that they found no significant harm. 

El-Tellawy et al. [30] reported on their prospective, open-label randomized clinical trial that included 146 children. The investigators used 1.5 ATA and 100% oxygen (40 sessions) combined or alone with sound therapy. Their primary outcome was the Childhood Autism Rating Scale (CARS). Both HBOT and good treatment independently demonstrated a significant benefit, 13.2% and 12% improvement in the CARS, respectively, over the control group, but the combination of HBOT and sound therapy was found to be the most beneficial (28.9% improvement). 

Kostiukow and Samborski [31] reported on 35 children with ASD receiving HBOT at 1.5 ATA without any control group and assessing the Clinical Global Impression Scale (CGIS), Autism Treatment Evaluation Checklist (ATEC), and CARS [31]. Although there was no control group, there was a statistical difference before/after in the ATEC and CARS, but younger children showed benefits in different categories than older children. Of the two recent publications, El-Tellawy et al. [30] would most likely have been included in the Cochrane Review, while Kostiukow and Samborski [31] would have been excluded due to the lack of any control group.

The risks of HBOT primarily involve middle ear or sinus barotrauma, but they may also include claustrophobia, anxiety, seizures, and reversible ocular-related changes [32,33]. Very few adverse effects have been reported in clinical trials. Reported side effects involve barotrauma, which does not require HBOT to be discontinued. Rossignol et al. reported that one child discontinued treatment due to worsening asthma symptoms [28]. Sampanthavivat et al. reported 11 instances of middle ear barotrauma, none of which required discontinuation of HBOT [26]. 

In our study, only the children who were administered VBMAPP demonstrated statistically significant improvements with a high ES. Those administered ABLLS had substantial improvement with a medium ES but a non-significant p = 0.2024 due to the low statistical power as the result of a high within-group variability between the control and experimental groups' difference scores.

One hypothetical explanation for this apparent discrepancy may be related to the patient’s age relative to the period of normative speech development and the efficiency of the curricular assessments. In this study, the mean age of children who were administered the VBMAPP was 4.1 years while the mean age of ABLLS participants was 7.8 years. The first three years of a child’s life are the most critical time for acquiring speech and language systems. During this time, sensory input stimulates brain synapse repeatedly until these connections become “hard-wired.” It becomes an efficient, permanent pathway that allows signals (that are later used for verbal behavior) to be transferred quickly and accurately. Advances in brain imaging technology have been used to verify this. The lack of intervention prior to five years of age has resulted in a significant loss of essential synapse connections needed in verbal speech. This “use it or lose it” mentality could account for the differences in benefit between the groups [34,35,36]. 

Another hypothesis that may account for the differences between each of the treatment groups is the malleability of the brain during different stages of life. Throughout child neurodevelopment, there are critical periods of time where brains are remarkably malleable. Developing brains are thought to possess the most plasticity for language development at three years of age [34,35]. As time passes, these “critical periods” begin to close, preventing plasticity as the brain reaches adulthood [36]. Patients who were administered the VBMAPP were on average 1.1 years outside this critical period, while those who received the ABLLS were on average 4.8 years outside the critical period. The longer the participant's brain is outside the critical period of malleability, the slower the participant may be to develop new verbal speech. 

Finally, the efficiency of the testing instruments could also account for the difference in the significant benefit between our treatment groups. The VBMAPP is based upon B.F Skinner’s analysis of verbal behavior, developmental milestones, and field-tested data from typically developing children and children with autism [12]. The VBMAPP is the most efficient tool and only measures verbal behavior. It is compiled of 170 individual milestones all relating to language development. The ABLLS assessment contains a task analysis of 25 different treatment areas and only 10 of which directly relate to language development [14]. 

This study had limitations that need pointing out. This study may not be representative of the entire children with ASD population, and it is difficult to detect differences between groups and to adjust for potential confounders fully. Of the 65 children, 43 (66.2%) were on one or more treatment therapies for ASD at the start of the cohort (other than hyperbaric treatment therapy). During the study, 22 children had a change in their original treatment therapy (62.9%), and seven had a stop in their initial treatment therapy (20.0%). Of those children, more experienced a change (81.0%) or stop in treatment (28.6%) in the HBOT group than in the non-HBOT group (35.7% and 7.1%, respectively). 

Eleven children who were not on therapy at baseline started a new therapy during the cohort period (six in the hyperbaric treatment cohort and five in the non-hyperbaric cohort). Eleven children were on one or more supplements at baseline and continued supplements through the cohort period. At baseline, three children were on one or more prescription medications in the non-HBOT group and none in the HBOT group. All three children also had additional prescription medications added to their regimens during the cohort period. 

This was a study of children with ASD for whom their parents self-selected for HBOT or not based on personal preferences, opinions, or financial means. As such, parents and families motivated to participate in HBOT may also have been providing additional therapies for their children.

There was no independent, objective confirmation of ASD diagnosis in each patient to ensure consistency with current DSM-V criteria. When the data were collected, the individuals documenting the VBMAPP and ABLLS may or may not have been aware of the patient’s participation with HBOT, which may have influenced the results. Children with ASD typically receive several treatments, both traditional and complementary. 

Although all patients carried a diagnosis of ASD as supplied by an outside medical provider, there was no independent, objective confirmation of this diagnosis in each patient to ensure consistency with the current DSM-V criteria. When the data were collected, the individuals documenting the VBMAPP and ABLLS may or may not have been aware of the patient’s participation with HBOT, which may have influenced the results. Children with ASD typically receive several treatments, both traditional and complementary.

## Conclusions

This study suggests a productive and safe effect of HBOT to improve verbal behaviors in a sample of children with ASD. Our study is unique in that it is the first to specifically look at the effects of HBOT on verbal behavior as measured by VBMAPP and ABLLS in children with ASD. Noteworthy mean differences were reported between the control (ABA only) and the experimental (ABA + HBOT) groups for VBMAPP and ABLLS pretest and posttest differences, with small to medium ESs for ABLLS to high ESs for VBMAPP.

Given that HBOT has demonstrated potential positive effects on a wide variety of general health outcomes, more specifically with verbal communication milestones in ASD children, our study validates efficacious HBOT outcomes relative to verbal behavior milestones, such as requesting, naming, or identifying objects, actions, and events, answering questions or having conversations, following instructions and complying with others, repeating what is heard, copying someone else's motor movements, reading written and writing words, motor skills, self-help skills, academic levels, social interaction, and language and communication skills.

Regardless of the positive results of this study, ongoing research is required as the effects of HBOT on verbal behavior remain inconclusive and may vary on the individual HBOT protocol and individual differences in the characteristics of sample subjects. TOC, the current site of this trial, remains one of 142 registered HBOT sites globally on ClinicalTrial.gov, conducting ongoing treatment and data collection with HBOT interventions. 

Our BCBAs sparked the interest in this study. They recognized a pattern of needing to create twice as many goals for the children who were undergoing HBOT compared to their peers. Similarly, many parents reported that their children spoke or sang for the first time after HBOT. The research staff was able to validate the anecdotal findings.

This study did provide insight and trends to examine further the impact of HBOT in children with autism, improving communication skills necessary for their development, behavior, learning, and socializing. The quality of relationships strengthens when both parties can express their feelings and ask for what they want. Acquiring verbal skills will make a significant difference for individuals with ASD. When communicating is a challenge, interacting with other people can be overwhelming. Improving the skills needed to participate in personnel and professional circles makes the individual’s experiences less frequently perceived as unfavorable. The prospect of job opportunities, romantic relationships, and independent living increases with the ability to express oneself.

As the results indicate, early intervention is critical to a successful outcome. However, children cannot benefit from the therapy if it is never presented as an option. Providers and parents are generally unaware that HBOT is a safe complementary adjunctive treatment. Oxygen may be the key to reducing inflammation and regulating the mitochondrial function required for optimal language acquisition.

The authors emphasize that, based on the impactful improvements in verbal behavior as measured by the VBMAPP and ABLLS inventories reported in this study, ongoing treatments and research are required to further demonstrate efficacy of HBOT as a viable intervention in the treatment of verbal behavior in children with autism.
