# Correction: The Effects of Hyperbaric Oxygen Treatment on Verbal Scores in Children With Autism Spectrum Disorder: A Retrospective Trial

**DOI:** 10.7759/cureus.c248

**Published:** 2025-08-13

**Authors:** Tami Peterson, Robert Sherwin, Tiffany Hosey, Nicole Close, Frederick Strale Jr.

**Affiliations:** 1 Hyperbaric Oxygen Therapy, The Oxford Center, Brighton, USA; 2 Hyperbaric Oxygen Therapy, Wayne State University School of Medicine, Detroit, USA; 3 Biostatistics, EmpiriStat, Kitty Hawk, USA; 4 Biostatistics, The Oxford Center, Brighton, USA

The Conflicts of Interest statement has been updated to declare the following:

Financial relationships: Tami Peterson, Robert Sherwin, Tiffany Hosey and Frederick Strale Jr. declare employment from The Oxford Center.

